# Influenza-Like-Illness and Clinically Diagnosed Flu: Disease Burden, Costs and Quality of Life for Patients Seeking Ambulatory Care or No Professional Care at All

**DOI:** 10.1371/journal.pone.0102634

**Published:** 2014-07-17

**Authors:** Joke Bilcke, Samuel Coenen, Philippe Beutels

**Affiliations:** 1 Centre for Health Economics Research & Modelling Infectious Diseases (CHERMID), Vaccine & Infectious Disease Institute (VAXINFECTIO) WHO collaborating Centre, Faculty of Medicine & Health Sciences, University of Antwerp, Antwerp, Belgium; 2 Centre for General Practice, Primary and Interdisciplinary Care (ELIZA), Faculty of Medicine & Health Sciences, University of Antwerp, Antwerp, Belgium; 3 Laboratory of Medical Microbiology, Vaccine & Infectious Disease Institute (VAXINFECTIO) WHO collaborating Centre, Faculty of Medicine & Health Sciences, University of Antwerp, Antwerp, Belgium; 4 School of Public Health and Community Medicine, Sydney, Australia; Fondazione Bruno Kessler, Italy

## Abstract

This is one of the first studies to (1) describe the out-of-hospital burden of influenza-like-illness (ILI) and clinically diagnosed flu, also for patients not seeking professional medical care, (2) assess influential background characteristics, and (3) formally compare the burden of ILI in patients with and without a clinical diagnosis of flu. A general population sample with recent ILI experience was recruited during the 2011–2012 influenza season in Belgium. Half of the 2250 respondents sought professional medical care, reported more symptoms (especially more often fever), a longer duration of illness, more use of medication (especially antibiotics) and a higher direct medical cost than patients not seeking medical care. The disease and economic burden were similar for ambulatory ILI patients, irrespective of whether they received a clinical diagnosis of flu. On average, they experienced 5–6 symptoms over a 6-day period; required 1.6 physician visits and 86–91% took medication. An average episode amounted to €51–€53 in direct medical costs, 4 days of absence from work or school and the loss of 0.005 quality-adjusted life-years. Underlying illness led to greater costs and lower quality-of-life. The costs of ILI patients with clinically diagnosed flu tended to increase, while those of ILI patients without clinically diagnosed flu tended to decrease with age. Recently vaccinated persons experienced lower costs and a higher quality-of-life, but this was only the case for patients not seeking professional medical care. This information can be used directly to evaluate the implementation of cost-effective prevention and control measures for influenza. In particular to inform the evaluation of more widespread seasonal influenza vaccination, including in children, which is currently considered by many countries.

## Introduction

The possibility of widespread seasonal influenza vaccination of children is receiving more and more attention [1]–[3]. Indeed, in line with other countries, Belgium recently investigated the costs and benefits of a range of childhood and adult influenza vaccination options through economic evaluation [4]. To inform such an evaluation, data are needed on the costs and health related quality-of-life (QoL) associated with influenza. This is well documented for hospitalized patients and ambulatory patients in other countries (e.g. [5]–[8]), but we know of only one study that provided similar information for persons with influenza for which no professional medical care is sought [9].

In view of this, the current article presents disease characteristics, health care use, absenteeism, costs, QoL score and Quality-Adjusted Life-Years (QALY’s) lost related to influenza-like-illness (ILI) and clinically diagnosed flu in patients in Belgium seeking ambulatory care or no professional medical care at all (i.e. ‘community patients’). Additionally, we assess whether the direct costs, QoL score and QALY’s lost are influenced by age, gender, vaccination status and having an underlying illness. Potential influential aspects can then be accounted for in evaluations of options to treat or prevent flu and ILI in specific subgroups (e.g. children, immunocompromized persons) [4]. Furthermore, this is one of the few studies formally comparing the direct costs, QoL score and QALY’s between ILI patients with and without flu as clinical diagnosis. Often it is not straightforward to collect data on flu due to the non-specific symptomatology and a lack of systematic testing [1], [10], and therefore proxies are used (e.g. acute respiratory illness, ILI). This study compares data on ILI and clinically diagnosed flu.

In summary, this is one of the first studies to (1) describe the out-of-hospital burden of clinically diagnosed flu and influenza-like-illness (ILI), also for patients not seeking professional medical care, (2) assess influential background characteristics, and (3) formally compare the burden of ILI patients with and without flu as clinical diagnosis.

## Methods

### Survey methods and participation

Between January and March 2012 roughly 10000 Belgian telephone numbers were dialled by random digit dialling on mobile and landlines. About half (4537 out of 9170) of the contacted persons (or someone in their household) complied with our definition for ILI (see below), and were asked to fill in a questionnaire about general and disease characteristics, health care use, absence from work or (pre-) school, and Quality-of-Life (QoL) (for the complete questionnaire, see Supporting Information S1). The respondents could choose to complete the questionnaire by phone, through the internet (they would then receive a survey-link by email within 10 minutes) or in writing (through the post services, with a pre-stamped return envelope), in both prevailing Belgian languages (Dutch and French). Consistency of recruitment protocols and surveys was verified by back translation. The questionnaire could be completed by the person who had ILI, or by a member of his/her household (e.g. parent of a sick child). Quality of Life assessment was based on the standardized SF-12v2 Health Survey, using Quality Metric Health Outcomes Scoring Software 4.5, for the responses in writing only.

We had resources available to collect 2250 eligible and complete questionnaires from people who experienced ILI. Incomplete returned questionnaires were excluded and replaced in accordance with the below quota to attain the sample size of 2250 (496 of them were replaced). The process of recruitment was following set quota targets, in order to approximate the gender specific population of the respective regions, in accordance with national statistics. Age quota were set as follows: <12 y (25%), 12–17 y (5%), 18–49 y (25%), 50–69 y (25%), >70 y (20%), to allow exploring age-specific cost differences and making sure robust estimates could be made for the most vulnerable in the population (i.e. by oversampling children under 12 years and the elderly). Hence, the age distribution of the sample was intentionally not representative for the complete (ILI) population, and we accounted for this by adding age as a covariate in all analyses (see further). The use of quota implies respondents were excluded if their background did not comply with the sought after characteristics at the time of sampling. To obtain the sample size of 2250, preset recruitment period (December up to mid February 2012) was extended up to March. It is noteworthy that the quotas were met at the end of the first recruitment period (mid February), meaning that the age and gender distribution of the respondents does not differ between the two periods of recruitment.

### Ethics statement

The Committee for Medical Ethics reviewed the study protocol, the questionnaire and the information letter for participants, and approved them 20th January 2014 (a posteriori). Data were analysed anonymously. Verbal informed consent was obtained from all persons that complied with our inclusion criteria and agreed via telephone to participate in the survey ( = verbal consent). Verbal consent is sufficient, as the survey was conducted without any physical or psychological intervention.

### Definitions of ILI and clinically diagnosed flu

All respondents are considered as having had influenza-like-illness (ILI), defined as having experienced at least 3 of the following 9 symptoms during the previous 4 weeks: rapidly rising fever, high fever (>38°C for an adult and >38.5°C for a child), sore throat, runny or blocked nose, cough, sore muscles, shivering, nauseous or vomiting, and tired or exhausted. Respondents who consulted a medical doctor were asked whether they received an explicit diagnosis from their doctor, and if so to specify this diagnosis in the questionnaire. To do this, they could choose between following options: flu or influenza, cold or bronchitis, pharyngitis, otitis, pneumonia, ‘other, namely …’, or ‘don’t know/remember’. Next, the respondents were classified by one of the authors (Medical Doctor S. Coenen) into three groups: likely flu, unlikely flu, or possibly flu (in case of doubt) as follows: (1) respondents who reported as diagnosis in the questionnaire only ‘flu or influenza’ were classified as ‘likely flu’, (2) respondents who reported only cold or bronchitis, pharyngitis, otitis and/or pneumonia were classified as ‘unlikely flu’, (3) respondents who reported more than one diagnosis and/or specified a specific diagnosis under ‘other, namely …’ were classified by SC under one of the three groups, depending on the reported diagnoses and their age. In sensitivity analysis, the impact of this classification on the results was assessed (see below).

### Estimating costs

The direct medical costs related to ILI are estimated by combining health care use data from the survey with the unit costs of the different types of physician consultations and medication. Unit costs for consultations are taken from the Belgian reimbursement scheme (www.riziv.be, accessed February 2012). The unit price for each medication is derived from the official price lists (www.bcfi.be, accessed May 2012). Data assumptions are limited as much as possible (for details see Supporting Information S2, and Beutels et al [4], p. 67–71), for instance the standard fees for regularly insured patients are used (which is according to Belgian guidelines [11]). To account for uncertainty, the highest and lowest unit price for each type of medication is used. This approach to acknowledge uncertainty was agreed upon in consultation with a group of experts (see acknowledgements). The total direct medical costs outside of hospital for an ILI episode include the costs of medication purchased and the costs of consultations (costs paid by the National Health care System (NHS) as well as co-payments by patients and their private insurers). These costs are calculated separately for community patients, ambulatory patients (including 18 patients with an emergency visit and/or an outpatient consultation in a hospital) and hospitalized patients.

### Statistical analysis

General linear models are used to assess (1) which of the background characteristics of the respondents, if any, influences the direct costs, QoL score and Quality-Adjusted Life-Years (QALY’s) lost of (a) ILI patients (i.e. all respondents) and (b) ILI patients with clinically diagnosed flu (‘likely flu’); and (2) whether the average direct costs, QoL score and QALY’s lost differ significantly between ambulatory ILI respondents categorized as ‘likely flu’ or as ‘unlikely flu’. The second analysis includes only interactions with background characteristics found significant in the first analysis. The best-fitting distributions for the response variables are used (for details see Tables S1 and S2). Models containing only significant (interactions of) covariates are retained, based on removing non-significant covariates (p>0.05, backward selection) and on likelihood ratio tests for dropping covariates.

Analyses are done using the highest and lowest cost as a response. In sensitivity analysis we investigated the impact of excluding the 18 patients with an emergency and/or hospital day consultation, of categorising the ‘possibly flu’ respondents as either ‘likely flu’ or ‘unlikely flu’, of excluding outliers and censored respondents. The cost and/or QoL per respondent is right-censored if the respondent was still sick at the moment the questionnaire was completed, and/or the cost for all medication taken and/or bought could not be determined because of lack of information (e.g. a person specified ‘other medication’ to be taken and bought, but gave no information on the type of medication group; medication was specified to be taken, but not clear if it was bought (or vice versa)).

Data management and analysis is done using the statistical software R (http://www.r-project.org/, packages stats and gamlss).

## Results

We collected information on 2250 persons who experienced ILI, with 435 of the ones seeking professional medical care categorised as ‘likely flu’. Table 1 presents background and disease characteristics, absenteeism, medical costs and QoL categorised by type of health care used, for all respondents, as well as for respondents classified as ‘likely flu’.

**Table 1:** Overview of background characteristics, disease burden, ambulatory care costs and Quality-of-life separately for all ILI patients and the ones clinically diagnosed as flu, according to type of health care used.

	COM, ILI(n = 1107)	AMB, ILI(n = 1116)	AMB, FLU(n = 429)	HOSP, ILI(n = 24)	HOSP, FLU(n = 6)
GENERAL					
media					
telephone	18%	24%	33%	17%	25%
written	45%	37%	28%	54%	50%
online	37%	38%	39%	29%	25%
gender					
male	48%	42%	43%	71%	50%
female	52%	58%	57%	29%	50%
age					
0–17	27%	33%	30%	33%	0%
18–64	51%	47%	51%	25%	25%
65 and older	22%	21%	19%	42%	75%
underlying illness	10%	16%	17%	38%	50%
pregnant$	1	10	4	0	0
previously vaccinated against flu	21%	20%	18%	33%	33%
between sept 2011 and feb 2012	12%	11%	9%	25%	33%
time of ILI episode					
december 2011	25%	21%	16%	25%	25%
january 2012	41%	35%	24%	33%	50%
february 2012	24%	32%	46%	38%	25%
march 2012	10%	11%	14%	4%	0%
DISEASE BURDEN					
number of symptoms|	4 (4) [3]–[9]	5 (5) [3]–[9]	6 (5) [3]–[9]	5 (5) [3]–[9]	5 (5) [3]–[7]
duration of symptoms (days)	6 (4) [1–50]	6 (5) [1–60]	6 (5) [2–30]	9 (7) [2]–[20]	9 (9) [3]–[15]
% of respondents who reported:					
rapidly rising fever	15%	30%	43%	29%	33%
high fever	15%	31%	44%	67%	17%
sore throat	12%	65%	64%	54%	33%
runny or blocked nose	60%	75%	70%	54%	50%
cough	82%	78%	76%	88%	67%
sore muscles	70%	51%	66%	46%	33%
shivering	37%	49%	60%	50%	17%
nauseous/vomiting	42%	34%	44%	46%	17%
tired and exhausted	21%	79%	86%	88%	50%
Diagnoses					
flu	NA	43%	100%	21%	100%
cold or bronchitis	NA	28%	0%	11%	0%
pharyngitis	NA	10%	0%	0%	0%
otitis	NA	2%	0%	5%	0%
pneumonia	NA	4%	0%	21%	0%
other	NA	14%	0%	42%	0%
% of respondents who tookany medication	66%	86%	91%	79%	100%
number of medication groups taken	2 (2) [0–8]	3 (3) [0–8]	3 (3) [0–8]	3 (3) [0–7]	3 (2) [2]–[4]
% of respondents who took medication:					
against fever	28%	42%	50%	39%	25%
against pain	30%	35%	38%	48%	25%
anti-inflammatory	14%	29%	32%	35%	75%
antibiotics	4%	45%	38%	52%	0%
anti-virals	5%	8%	15%	13%	50%
against cough	41%	48%	52%	26%	25%
against sore throat	38%	39%	39%	43%	0%
nose spray	45%	45%	45%	39%	5%
other	9%	16%	12%	17%	0%
Number of medical visits:	NA	1.6 (1) [0–19]	1.6 (1) [0–13]	3.7 (3) [0–10]	1.8 (2) [1]–[2]
GP consults	NA	1.1 (1) [0–6]	0.97 (1) [0–4]	1.0 (0.5) [0–4]	0.5 (0) [0–2]
GP home visits	NA	0.3 (0) [0–6]	0.4 (0) [0–6]	0.7 (0) [0–5]	0.8 (0.5) [0–2]
specialist consults and home visits	NA	0.2 (0) [0–12]	0.2 (0) [0–7]	2.0 (1.5) [0–9]	0.5 (0) [0–2]
% of consultations with GP	NA	84%	86%	45%	71%
Number of medical visits children (n = 671):	NA	1.7 (1) [1]–[19]	1.5 (1) [7–7]		
Interrupted normal daily activitiesdue to ILI	34%	72%	85%	88%	100%
for respondents reporting absence#:					
days’ rest	3 (2) [1]–[15]	5 (4) [1–30]	5 (5) [1]–[23]	8 (5) [1–27]	3.5 (3) [3]–[5]
work (days)	2 (2) [1]–[9]	4 (3) [1]–[21]	4 (4) [1]–[21]	2 persons: 14and 15 days	NA
school (days)	2 (2) [1]–[5]	4 (4) [1]–[20]	4 (4) [1]–[14]	2 persons: 2and 8 days	NA
kindergarten (days)	2 (2) [1]–[2]	5 (3.5) [2]–[10]	2 persons: 3and 10 days	NA	NA
Someone else interruptednormal daily activities tocare for ILI person	6%	15%	17%	46%	NA
for respondents reportinginterruption from someone else:					
number of days interrupted	2 (2) [1]–[10]	3 (3) [1]–[14]	4 (3) [1]–[14]	6 (5) [2]–[21]	NA
number of days interruptedto care for ILI children	3 (2) [1]–[10]	3 (3) [1]–[14]	4 (3) [1]–[14]	5 (5) [2]–[8]	NA
COSTS					
consultations					
low	NA	€39 (20) [0–551]	€39 (20) [0–435]	€101 (72) [0–292]	€52 (52) [35–70]
high	NA	€43 (23) [0–595]	€43 (23) [0–444]	€112 (76) [0–338)	€55 (58) [35–70)
medication					
low	€4 (0) [0–50]	€13 (10) [0–62]	€14 (11) [0–62]	€18 (19) [0–70]	€20 (17) [6–39]
high	€7 (0) [0–81]	€21 (15) [0–93]	€23 (23) [0–93]	€28 (27) [0–107]	€28 (22) [14–54]
total					
low	€3 (0) [0–50]	€51 (41)[0–597]	€53 (43) [5–477]	€120 (98) [6–313]	€72 (69) [41–109]
high	€7 (0) [0–81]	€64 (56) [€0–684]	€67 (58) [12–521]	€140 (112)[13–368]	€83 (80) [49–125]
QUALITY OF LIFE (written responses only)					
Quality-of-life score	0.70 (0.66)[0.47–1]	0.68 (0.66)[0.37–1]	0.68 (0.66)[0.48–0.92]	0.61 (0.64)[0.38–0.74]	0.62 (0.62)[0.58–0.66]
Quality-Adjusted Life-Yearslost	0.005 (0.004)[0–0.047]	0.006 (0.005)[0–0.075]	0.005 (0.005)[0–0.032]	0.009 (0.008)[0.002–0.019]	0.009 (0.008[0.003–0.017])

COM = community patients (not seeking professional medical care), AMB = ambulatory patients, HOSP = hospitalized patients, ILI = influenza-like illness, GP =  general practitioner. For continuous variables mean (median) [minimum-maximum] are reported.

$ ranging from 1 up to 7 months pregnant (3 of them vaccinated against flu).

| Minimum is 3: respondents were included only if they experienced at least 3 symptoms.

# Absence from work, school and kindergarten: responses are matched according to age (kindergarten [0–3], school [2.5–18], and work [18 and older), to correct for misspecified responses (47 records, e.g. specifying absence from kindergarten, school ánd work for a sick child).

### Influenza-like-illness and influential aspects

The proportion of all respondents seeking professional medical care was higher in the influenza peak incidence months, in February and March (57% and 52%), than in December and January (46% and 45%). Patients seeking medical care reported more symptoms (especially more often fever), a longer duration of symptoms, used more medication more often (including antibiotics), and reported more frequent interruption of normal activities as opposed to community patients. Patients seeking ambulatory medical care lost on average 4 to 5 days of kindergarten, school or work compared to only 2 days for community patients. Almost half of the patients seeking ambulatory medical care were diagnosed with flu only. They consulted 5 times more often a general practitioner (GP) than a specialist. Hospitalized patients were mostly diagnosed with flu, pneumonia or something else than bronchitis, pharyngitis or otitis, and consulted more often a specialist than a GP. The average direct cost for ILI treatment per respondent is mostly attributable to the cost for consultations, and ranged from less than €10 (community patients), to €50–€65 (ambulatory care), up to €120–€140 (out-of-hospital costs for hospitalized respondents). The QoL score was 0.70, 0.68 and 0.61 for community, ambulatory and hospitalized patients, resulting in 0.005, 0.006 and 0.009 QALY’s lost per episode, respectively. These results are presented in Table 1.

Results of the statistical analyses are shown in Table 2 and Table S1. The older the ILI community patient, the higher the probability to have a non-zero cost (Table 2). They report significantly higher non-zero costs and lower QoL when not vaccinated in 2011–2012 and when having an underlying illness (Table 2). Ambulatory ILI patients with an underlying illness report significantly higher costs and lower QoL scores and QALY’s lost than the ones without an underlying illness (Table 2). The older the ambulatory ILI patient, the higher the reported QALY’s lost. These results are robust in sensitivity analyses (Table S1).

**Table 2:** Estimated direct medical cost and quality-of-life associated with ILI and clinically diagnosed flu patients in Belgium, as a function of significant predictor variables (gender, underlying condition ‘cond’, age and/or vaccination status (‘vac’ = vaccinated just before or during the last flu season)).

group ofrespondents	response variable	significant predictor variables			model estimate for theresponse variable
community, ILI	probability to have no costs			age# = 5	76% [71–80%]
				age = 50	63% [60–66%]
				age = 50	56% [50–61%]
	average cost if not zero,lowest value		no cond	not vac	10 € [9]–[11]
				vac	8 € [6]–[10]
			cond	not vac	13 € [9]–[16]
				vac	10 € [7]–[13]
	average cost if not zero,highest value	men	no cond	not vac	20 € [18]–[22]
				vac	16 € [13]–[19]
			cond	not vac	26 € [21–32]
				vac	21 € [16–26]
		women	no cond	not vac	21 € [20]–[23]
				vac	17 € [14]–[21]
			cond	not vac	28 € [22–35]
				vac	23 € [17–29]
	quality-of-life score		no cond	not vac	0.70 [0.69–0.71]
				vac	0.73 [0.71–0.76]
			cond	not vac	0.64 [0.62–0.67]
				vac	0.67 [0.64–0.70]
	Quality-adjusted life-yearslost		no cond		0.0045 [0.0040–0.0050]
			cond		0.0059 [0.0048–0.0071]
ambulatory, ILI	average cost, lowest value		no cond	age# = 5	52 € [48–57]
				age = 50	48 € [46–50]
				age = 75	46 € [44–49]
			cond	age# = 5	70 € [60–81]
				age = 50	63 € [56–72]
				age = 75	60 € [52–70]
	average cost, highest value		no cond		62 € [60–65]
			cond		78 € [69–87]
	quality-of-life score				0.68 [0.67–0.69]
	Quality-adjusted life-yearslost		no cond	age# = 5	0.0044 [0.0038–0.0052]
				age = 50	0.0056 [0.0050–0.0063]
				age = 75	0.0069 [0.0052–0.0073]
			cond	age# = 5	0.0073 [0.0060–0.0089]
				age = 50	0.0086 [0.0074–0.0099]
				age = 75	0.0099 [0.0078–0.0107]
ambulatory, likelyflu	average cost, lowest value		no cond		48 € [45–51]
			cond		79 € [64–98]
	average cost, highest value		no cond		61 € [58–65]
			cond		94 € [76–113]
	quality-of-life score				0.68 [0.67–0.70]
	Quality-adjusted life-yearslost		no cond		0.0043 [0.0037–0.0050]
			cond		0.0075 [0.0061–0.0095]

95% uncertainty intervals are obtained by bootstrapping (1000 samples). #Age was included as continuous predictor variable in the regression models but for clarity this table presents estimates for only 3 ages (in years).

### ILI clinically diagnosed as flu versus other ILI (ambulatory care)

‘Likely flu’ patients reported more symptoms (on average 6 instead of 5) than all 2250 ILI respondents, especially more often fever. A larger proportion of the ‘likely flu’ respondents took medication, in particular more often antivirals, which results in a higher average cost for medication. Although the total number of doctor visits does not differ significantly between ‘likely flu’ and ‘unlikely flu’ patients (Wilcoxon test, p = 0.30 (all respondents), p = 0.40 (children only (<18 yrs))), ‘likely flu’ patients more often consult a specialist (instead of a GP) or a GP at their home (instead of at the GP’s practice). The number of days someone interrupts their daily activities to care for a ‘likely flu’ patient is not significantly different from the number of days for an ‘unlikely flu’ patient (Wilcoxon test, p = 0.57 (all respondents), p = 0.40 (children only)). The proportion of respondents classified as ‘likely flu’ is larger in February and March than in December and January. The subgroup of hospitalized patients was too small to make reliable comparisons about their specific out of hospital experience (6 respondents categorized as ‘likely flu’). These results are presented in Table 1.

**Figure 1:**
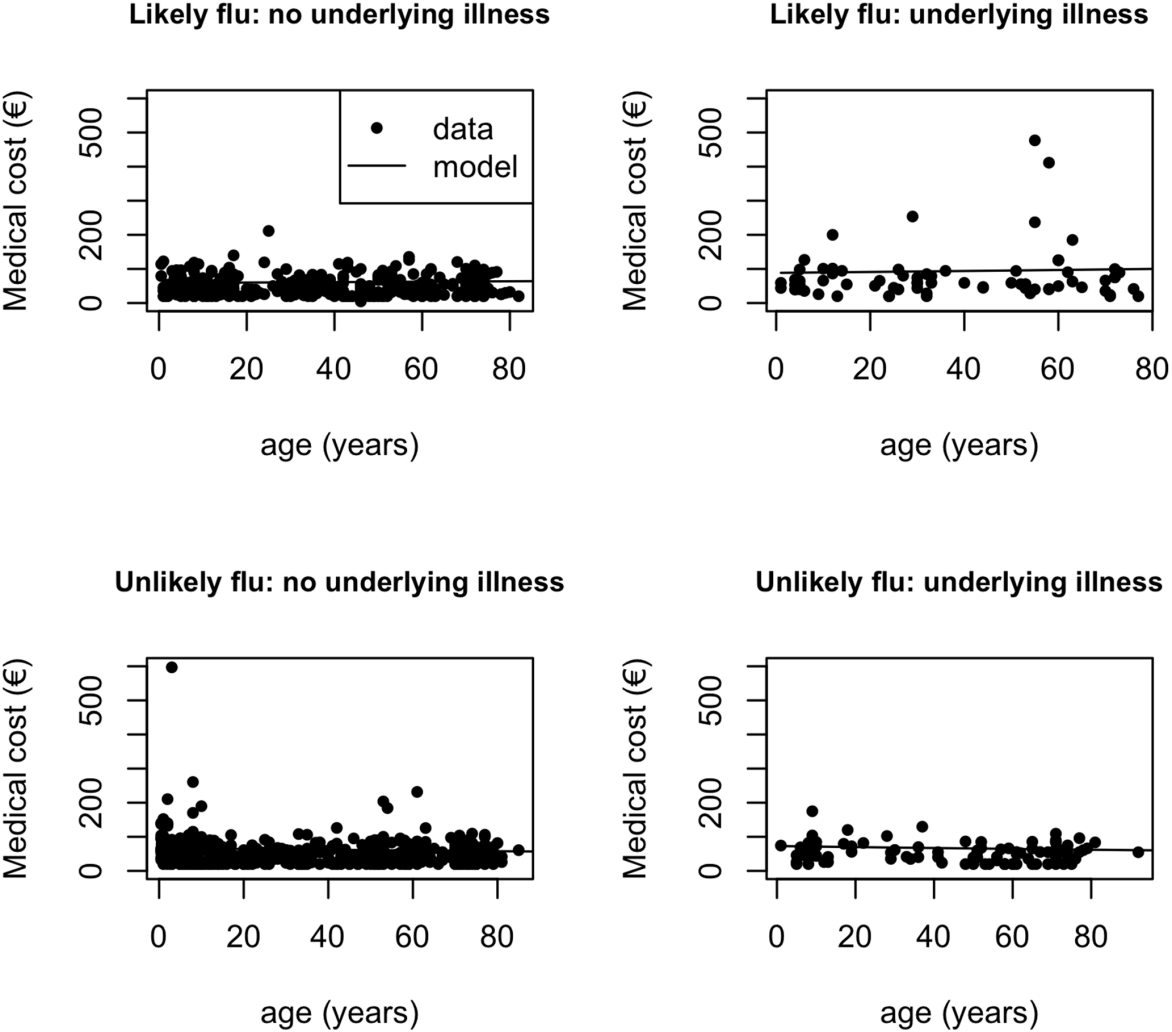
Costs for ambulatory ILI patients. Estimated average direct medical cost (€, using lowest unit cost for medication) as a function of having an underlying illness and age, separately for ambulatory ILI respondents categorized as ‘likely flu’ or ‘unlikely flu’.

**Figure 2:**
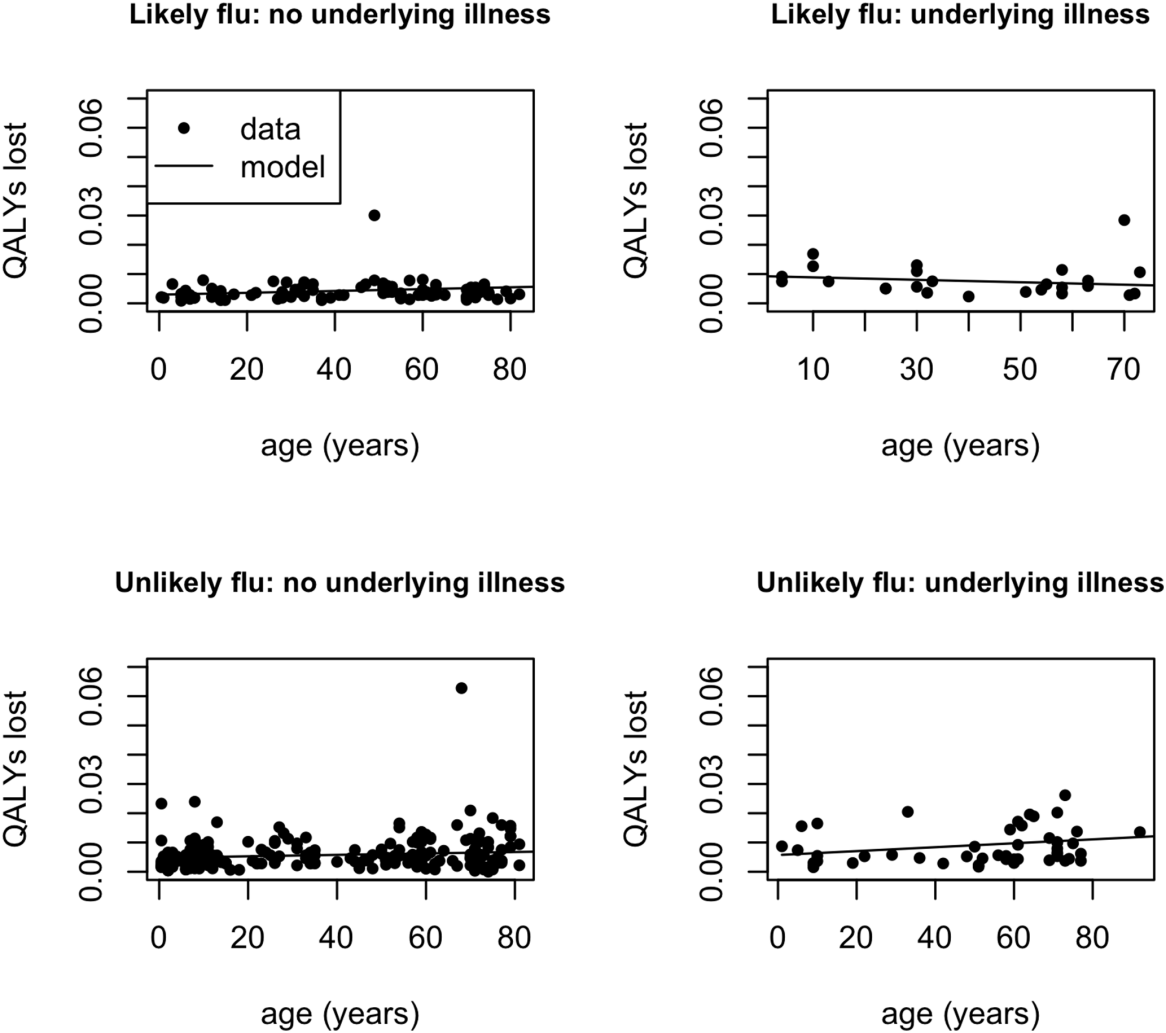
Quality-Adjusted Life-Years lost for ambulatory ILI patients. Estimated average Quality-Adjusted Life-Years lost as a function of having an underlying illness and age, separately for ambulatory ILI respondents categorized as ‘likely flu’ or ‘unlikely flu’.

Results of the statistical analyses are shown in Figure 1 and Figure 2 and Table S2. For patients with an underlying illness, those categorised as ‘likely flu’ incur on average higher costs than those categorised as ‘unlikely flu’. This is not the case for patients without underlying illness (Figure 1). The relationship between cost and age differs significantly between ‘likely flu’ patients (increasing trend) and ‘unlikely flu’ patients (decreasing trend) (Figure 1). This difference is no longer significant when the three respondents with the largest costs (>€400) are excluded (Table S2). The QALY’s lost are significantly higher for ‘unlikely flu’ patients than for ‘likely flu’ patients without an underlying illness (Figure 2). The relationship between QALY’s lost and age differs significantly between ‘likely flu’ patients (decreasing trend) and ‘unlikely flu’ ILI patients (increasing trend) with an underlying illness (Figure 2). The QoL score does not significantly differ between these two categories (0,68 for both groups). These results are robust in sensitivity analysis (Table S2).

## Discussion

Our study is the first for Belgium to give an extensive overview of the disease characteristics, costs, QoL score and QALY’s lost related to both ILI and clinically diagnosed flu, for ambulatory patients and for patients not seeking professional medical care.

As expected, patients seeking medical care report a higher cost and a lower quality of life than community patients. Having an underlying illness strongly increases the average cost and decreases the average quality of life for all ILI patients. Although previous vaccination in the same season is associated with lower cost and better quality of life for community patients, this is not the case for ambulatory care patients. Possibly, this group includes people who did not mount a sufficient immune response after vaccination (primary vaccine failure) and/or patients who were vaccinated only very shortly before the onset of disease. Cost and quality-of-life is very similar for ILI patients whether or not they were clinically diagnosed with flu, except when they have an underlying illness. The decreasing trend in costs by age for ILI patients categorized as ‘unlikely flu’, could be explained by the relatively higher proportion of children with pneumonia. They reported higher costs than respondents with any of the other physician diagnoses.

The proportion of respondents classified as ‘likely flu’ was larger in February and March than in December and January. Also, the proportion of all respondents that sought professional medical care was higher in February and March (57% and 52%) than in December and January (46% and 45%). In view of the time-specific incidence of flu, which peaked during February and March during the 2011–2012 season, this could indicate that flu patients are more likely to seek medical care. However this cannot be tested with this dataset, since we do not know which community ILI patients had laboratory confirmed flu.

Overall, the medication use, costs and absenteeism associated with clinically diagnosed flu in Belgium are very similar to what has been reported for other West-European countries [12]. Similar to our study, Principi and Esposito [13] did not find a difference in number of medical visits between flu positive and flu negative children. However, an Italian study [14] reports a much higher average cost for patients with flu or ILI than in our study, as well as 32% higher costs for flu patients compared to ILI patients without flu. They found higher costs for flu patients because compared to parents of ILI patients without flu, the mother and father incurred 1 and 2 more work days lost, respectively [14]. In our study, there was no significant difference between ‘likely flu’ and ‘unlikely flu’ children with respect to the number of days someone else had to interrupt their normal activities to provide care. When considering only the ambulatory direct medical costs, the average cost of the Italian study reduces to about €38 for both flu positive and flu negative patients, which is 10 to 20 euros less than what we found in our study. Additionally, they did not find these direct costs to increase with age [14]. Antonova and colleagues [12] found no age trend in medication use in children, but reported fewer medical visits for flu in older children as compared to infants and toddlers. However, the latter observation can also be due to the fact that the infants and toddlers were recruited from an emergency department [15], [16], and not from a primary care setting like the older children [13].

**Figure 3:**
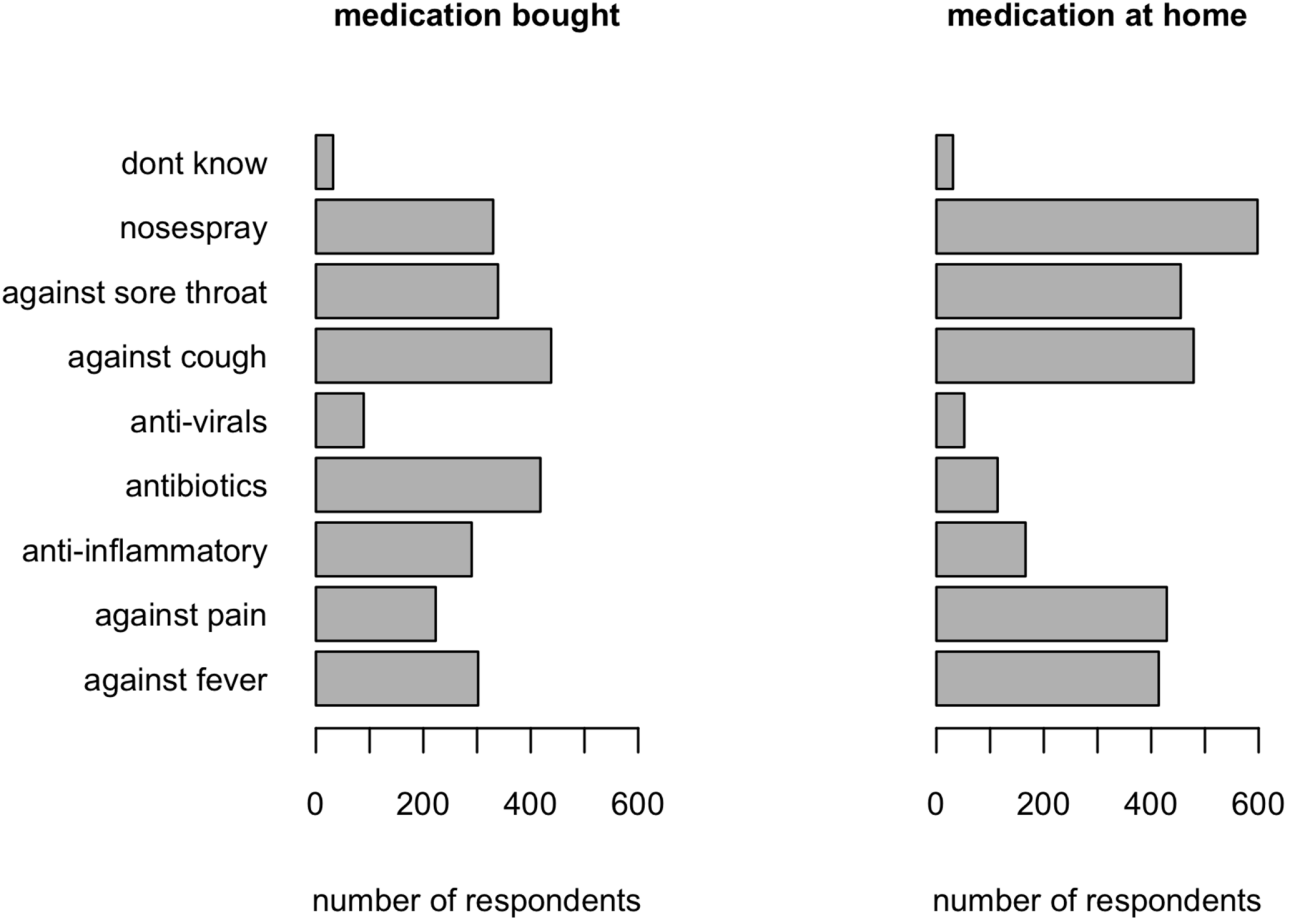
Medication use of ILI patients. Number of respondents using different types of medication, separately for medication bought in the pharmacy and medication that respondents had in storage at home. Each respondent could specify several types of medication bought or at home. ‘Don’t know’ refers to medication taken without being sure about which type.

It is astonishing how much ‘prescription only’ medication, people have at their disposal in their homes (Figure 3). This type of medication can only be obtained when specifically prescribed by a medical doctor. It is therefore unwanted that many persons have such medication at home, allowing them to take it whenever they see fit (rather than by physician’s advice). Yet 2% of the respondents, who did not consult a physician, took antibiotics for their ILI episode. This is about four times the rate that was reported in a previous general population sample in Belgium [17]. Antibiotic use for ILI was found to be much higher in primary care (45%). For comparison, Butler et al [18] reported almost 30% antibiotics use in adults with a new or worsening cough or clinical presentation suggestive of lower respiratory tract infection in Antwerp (Belgium). The benefit of antibiotics for an ILI like bronchitis is little [19]. Also, despite the modest benefit of antivirals [20], in this study they are used more than twice as often to treat ‘likely flu’ patients compared to ‘unlikely flu’ ILI patients in ambulatory care.

To our knowledge, this is the only study having used SF-6D as a measure for the QoL associated with ILI and clinically diagnosed flu retrospectively. We found a lower impact of flu on QoL than previous published studies (summarized in van Hoek et al [21]). Possibly, our study underestimated the impact of flu on the QoL because we used as definition for flu the clinical diagnosis of a medical doctor and not a laboratory test like van Hoek et al and Hollmann et al [21], [22]. Our study would underestimate the impact if ILI patients falsely diagnosed as having flu, generally experienced less of a burden than true flu patients.

Ours is the only study to date, which modelled the loss in QALY’s as a function of important covariates. As expected, having an underlying illness leads to more QALY’s lost. Also, the older the patient, the more QALY’s they lost (except for patients classified as ‘likely flu’ and having an underlying illness). This is in line with Sander et al [24], who found that older patients lost more QALY’s than younger patients. This age-related trend in our study is due to elderly people being sick for a longer time, because we did not find the QoL score to depend on age. Note however that we probably overestimated the QALY’s lost for elderly as compared to younger patients, because of the way we calculated the QALY’s lost. Namely, we assumed persons without flu to be in perfect health, irrespective of their age. This choice was made because currently no baseline QoL data exist for the Belgian population.

### Strengths and limitations of the methods used

In the absence of a clear-cut definition for ILI, we assumed a person with ILI to have at least 3 of a list of 9 symptoms. This definition is similar, but not the same as used in other studies (for example, the latest definition for ILI proposed by the World Health Organization is ‘an acute respiratory illness with a measured temperature of ≥38°C and cough, with onset within the past 7 days’, WHO_HSE_GIP_2011.1_eng.pdf, accessed 16 May 2013). Also, we categorized ILI patients as ‘likely flu’ based on the clinical diagnosis of a medical doctor, although ideally a lab test should be used systematically. Therefore, our results should be interpreted with care. However, it is comforting that our results are robust for different classifications of flu diagnoses (i.e. adding ‘possibly flu’ with ‘likely flu’ or ‘unlikely flu’), and that our results are comparable with previously published studies (see above).

It is very difficult to obtain information on the specific flu-related burden in community patients. Although we managed to survey persons with ILI who did not consult a physician, our study design did not allow assessing directly whether these patients had flu or some other ILI.

Large efforts were made to estimate the direct medical costs for each respondent accurately. This was straightforward for the costs related to consultations, but more difficult for medication costs. However we believe our estimates are reasonable, because we compared them with the responses to a separate question on the amount paid for medication in the pharmacy (nothing, €0–25, €25–50, €50–75, €75–100, or more than €100). The average cost based on this extra question lies in between the lowest and highest average costs we estimated in the first place.

The proportion of respondents that used each of the three media (telephone, online, written) differed according to health care use and diagnosis. Also, for community patients, we found slightly lower costs for surveys completed online than for surveys by telephone. For patients who sought medical care, the choice of medium made no difference (results not shown).

### Implications

Our study demonstrates that the duration of symptoms, type of symptoms and number of symptoms differ between ambulatory and community patients. This excludes the use of existing prediction rules (e.g. [25]) to estimate indirectly the proportion of community ILI patients with influenza. Such a prediction model would estimate the likelihood of having flu based on a combination of the patient’s background characteristics and symptoms. However, apart from the problems with sensitivity and specificity of prediction rules, most of these are based on ambulatory patients. Hence, large prospective studies in the community seem to be the only way to assess the disease burden of flu in persons not seeking professional medical care, keeping in mind that the participation in such a study could have an important impact on health care seeking behaviour of the subjects.

However, for the purpose of health economic evaluation, the difficulties in assessing whether a patient had flu or another influenza-like-illness may outweigh the benefits in terms of data accuracy, because the differences in costs and QoL for ambulatory care patients, as assessed by our study, are negligible. As such, this study provides estimates of cost and QoL related to clinically diagnosed flu and ILI, which can be used directly in analyses evaluating the cost-effectiveness of vaccination against seasonal influenza. Indeed, these estimates have been used to inform the cost-effectiveness of vaccinating specific target groups in Belgium, including children, health care workers, elderly and pregnant women [4]. Furthermore, this study indicates that different estimates for costs and QoL should be used, depending on the age of the target group for influenza vaccination, and on whether or not the target group includes a (large) proportion of persons with an underlying illness.

## Supporting Information

Table S1 **Models of direct medical cost and quality-of-life associated with ILI as a function of significant background characteristics.** The best-fitting distributions for the response variables are used^#^.
(DOCX)

Table S2 **Models and likelihood ratio tests to assess whether a clinical diagnosis of flu (yes or no) significantly influences the direct cost, Quality-of-Life score and Quality-Adjusted Life-Years (QALY’s) lost associated with ILI.** The best-fitting distributions for the response variables are used^#^.
(DOCX)

Supporting Information S1 **Questionnaire used to obtain information on the disease burden, costs and Quality-Adjusted Life-Years of persons who experienced influenza-like-symptoms, in Belgium.** This is a translation of the original Dutch and French questionnaires used for completion in writing. In the online and telephone questionnaires, respondents had for each question related to influenza-like-illness the option to answer ‘no answer’ (specified as one or more of the following: don’t remember/not sure/prefer not to answer/no opinion/no answer), because in the online system respondents had to give an answer to be able to continue with the next question. Persons could return to previous questions to specify a different answer. The online system was also used to record answers for the questionnaires completed by phone.
(DOCX)

Supporting Information S2 **Unit costs for different types of medication groups and consultations. (a)** Approach to derive the unit price for different types of medication from ‘het Gecommentarieerd Geneesmiddelen Repertorium/Répertoire Commenté des Médicaments’ (‘BCFI’, www.bcfi.be, accessed May 2012). The approach was agreed upon by a group of experts (S. Coenen and the expert committee of the KCE report 204, see acknowledgments). **(b)** Table: Unit costs for different types of medication groups and consultations (National Health System + co-payments, €2012).
(DOCX)
