# Supplementary material for: Influenza-Like-Illness and Clinically Diagnosed Flu: Disease Burden, Costs and Quality of Life for Patients Seeking Ambulatory Care or No Professional Care at All
Source: PLoS One. 2014 Jul 17;9(7):e102634. doi: 10.1371/journal.pone.0102634 (PMC4102549; doi:10.1371/journal.pone.0102634)
Supplement: Table S1 — Models of direct medical cost and quality-of-life associated with ILI as a function of significant background characteristics. The best-fitting distributions for the response variables are used#. (DOCX) [file pone.0102634.s001.docx]

**Influenza-Like-Illness and clinically diagnosed flu: disease burden, costs and Quality of Life for patients seeking ambulatory care or no professional care at all**

Joke BILCKE, Samuel COENEN, Philippe BEUTELS

**SUPPORTING TABLE S1: Models of direct medical cost and quality-of-life associated with ILI as a function of significant background characteristics.** The best-fitting distributions for the response variables are used^#^.

| response |  | | intercept | regression coefficients | | | | likelihood ratio test*^§^* | | |
| --- | --- | --- | --- | --- | --- | --- | --- | --- | --- | --- |
|  |  | |  | age | gender | cond | vac | χ² | df | p-value |
| Cost community patients (zero-inflated Poisson regression: coefficients on log (mu) or logit scale (sigma))^£^ | | | | | | | | | | |
| using lowest unit cost for medication: | | mu | 2.29 | NS | NS | 0.24 | -0.23 | 9.63 | 5 | 0.09 |
|  |  | p | 1.2 | -0.01 | NS | NS | NS |  |  |  |
| excluding censored records: | | mu | 2.32 | NS | NS | NS | -0.15 | 9.25 | 5 | 0.10 |
|  |  | p | 1.45 | -0.01 | -0.32 | NS | NS |  |  |  |
| using highest unit cost for medication: | | mu | 2.98 | NS | 0.08 | 0.27 | -0.21 | 5.56 | 4 | 0.23 |
|  |  | p | 1.2 | -0.01 | NS | NS | NS |  |  |  |
| excluding censored records: | | mu | 3.06 | NS | NS | 0.11 | -0.18 | 6.76 | 4 | 0.15 |
|  |  | p | 1.45 | -0.01 | -0.32 | NS | NS |  |  |  |
| Cost ambulatory care (gamma regression: coefficients on inverse scale) | | | | | | | | |  |  |
| using lowest unit cost for medication: | |  | 0.019 | 3e-5 | NS | -0.005 | NS | 2.79 | 2 | 0.25 |
| excluding censored records: | |  | 0.021 | NS | NS | -0.005 | NS | 3.33 | 3 | 0.34 |
| excluding outpatient records: | |  | 0.019 | 3e-5 | NS | -0.005 | NS | 3.06 | 2 | 0.22 |
| flu patients only: | |  | 0.021 | NS | NS | -0.008 | NS | 0.98 | 3 | 0.81 |
| using highest unit cost for medication: | |  | 0.016 | NS | NS | -0.003 | NS | 5.83 | 3 | 0.12 |
| excluding censored records: | |  | 0.017 | NS | NS | -0.003 | NS | 2.69 | 3 | 0.44 |
| excluding outpatient records: | |  | 0.016 | NS | NS | -0.003 | NS | 6.37 | 3 | 0.10 |
| flu patients only: | |  | 0.016 | NS | NS | -0.006 | NS | 1.60 | 3 | 0.66 |
| Quality-of-life community patients (normal regression, written responses only) | | | | | | | | | |  |
| quality-of-life score: | |  | 0.7 | NS | NS | -0.06 | 0.03 | 1.73 | 2 | 0.42 |
| excluding censored records: | |  | 0.7 | NS | NS | -0.06 | 0.03 | 0.08 | 2 | 0.96 |
| Quality-of-life ambulatory care (normal regression, written responses only) | | | | | | | | | |  |
| quality-of-life score: | |  | 0.68 | NS | NS | NS | NS | 4.90 | 4 | 0.30 |
| excluding censored records: | |  | 0.69 | NS | NS | -0.03 | NS | 3.4 | 3 | 0.33 |
| excluding outpatient records: | |  | 0.69 | NS | NS | -0.03 | NS | 1.75 | 3 | 0.63 |
| flu patients only: | |  | 0.68 | NS | NS | NS | NS | 4.45 | 4 | 0.35 |
| Quality-Adjusted Life-Years lost community patients (beta regression (mu) with dispersion (sigma): coefficients on identity (mu) or log (sigma) scale, written responses only) | | | | | | | | | |  |
| Quality-Adjusted Life-Years lost: | | mu | 0.0045 | NS | NS | 0.0014 | NS | 1.51 | 4 | 0.82 |
|  | | sigma | -2.94 | 0.01 | -0.26 | -0.46 | NS |  |  |  |
| excluding censored records: | | mu | 0.004 | 1e-5 | NS | 0.002 | NS | 0.15 | 2 | 0.93 |
|  | | sigma | -3.16 | 0.015 | -0.16 | -0.24 | -0.42 |  |  |  |
| Quality-Adjusted Life-Years lost ambulatory care (beta regression (mu) with dispersion (sigma): coefficients on identity (mu) or log (sigma) scale, written responses only) | | | | | | | | | |  |
| Quality-Adjusted Life-Years lost: | | mu | 4e-3 | 3e-5 | NS | 3e-3 | NS | 4.21 | 5 | 0.52 |
|  | | sigma | -3.12 | 0.01 | NS | NS | NS |  |  |  |
| excluding censored records: | | mu | 4e-3 | 2e-5 | NS | 2e-3 | 2e-3 | 0.26 | 3 | 0.97 |
|  | | sigma | -2.19 | NS | -0.85 | -0.36 | NS |  |  |  |
| excluding outpatient records: | | mu | 4e-3 | 3e-5 | NS | 3e-3 | NS | 4.45 | 5 | 0.49 |
|  | | sigma | -3.13 | 0.01 | NS | NS | NS |  |  |  |
| flu patients only: | | mu | 0.4e-3 | NS | NS | 3e-3 | NS | 11.93 | 7 | 0.10 |
|  | | sigma | -3.18 | NS | NS | NS | NS |  |  |  |

*p=mean probability to have no costs (i.e. cost=€0); mu=mean cost if having a non-zero cost or mean quality-adjusted life-years ‘s lost; sigma=dispersion parameter, it is used because the covariates have not only an effect on the mean, but also on the variance of the quality-adjusted life-years; NS=not significant (p>0.05); cond=underlying condition (no or yes); vac=vaccinated just before or during the last flu season.*

*^#^Best fitting distributions: the Gamma for costs of ambulatory patients (for a cost variable, distributions between 0 and infinity are appropriate, the Gamma distribution fitted better than Poisson, Lognormal and Negative Binomial distribution); the zero-inflated Poisson for costs of community patients, which is a mixture of a binary distribution (accounting for the many community patients with zero costs) and the Poisson distribution (reflecting the distribution of costs for respondents with expenses); the Normal distribution fitted well the QoL; and the beta distribution fitted well the QALY’s lost, as they were highly skewed but fell within the 0 to 1 interval.*

*^§^Likelihood ratio test compares model including all 4 covariates with model including significant covariates only.*

*^£^Only for the costs of community patients and quality-adjusted life-years, some of the interaction terms were found significant, however, the results were not consistent in sensitivity analysis. Possibly, the power is too low (when including 2- and 3-way interactions terms, sample size per group becomes small), that is why we only considered the models with main effects.*
