# Supplementary material for: Influenza-Like-Illness and Clinically Diagnosed Flu: Disease Burden, Costs and Quality of Life for Patients Seeking Ambulatory Care or No Professional Care at All
Source: PLoS One. 2014 Jul 17;9(7):e102634. doi: 10.1371/journal.pone.0102634 (PMC4102549; doi:10.1371/journal.pone.0102634)
Supplement: Table S2 — Models and likelihood ratio tests to assess whether a clinical diagnosis of flu (yes or no) significantly influences the direct cost, Quality-of-Life score and Quality-Adjusted Life-Years (QALY’s) lost associated with ILI. The best-fitting distributions for the response variables are used#. (DOCX) [file pone.0102634.s002.docx]

**Influenza-Like-Illness and clinically diagnosed flu: disease burden, costs and Quality of Life for patients seeking ambulatory care or no professional care at all**

Joke BILCKE, Samuel COENEN, Philippe BEUTELS

**SUPPORTING TABLE S2: Models and likelihood ratio tests to assess whether a clinical diagnosis of flu (yes or no) significantly influences the direct cost, Quality-of-Life score and Quality-Adjusted Life-Years (QALY’s) lost associated with ILI.** The best-fitting distributions for the response variables are used^#^.

| response |  | intercept | | regression coefficients | | | | | | | likelihood ratio test^§^ | | |
| --- | --- | --- | --- | --- | --- | --- | --- | --- | --- | --- | --- | --- | --- |
|  |  |  | | flu | cond | age | flu*cond | flu*age | cond*age | flu*cond*age | χ² | df | p-value |
| Cost ambulatory care (gamma regression: coefficients on inverse scale) | | | | | | | | | | | | | |
| using lowest unit cost for medication*: |  | 0.021 | | -3.2e-3 | -8.1e-3 | -1.7e-5 | 6.2E-3 | 7.0e-5 | NS | NS | 1.58 | 2 | 0.45 |
| excluding outliers#: |  | 0.021 | | -2.7e-4 | -6.2e-3 | NS | 4.4e-3 | NS | NS | NS | 5.92 | 4 | 0.21 |
| excluding censored records: |  | 0.021 | | NS | -5.0e-3 | NS | NS | NS | NS | NS | 5.11 | 6 | 0.53 |
| excluding outpatient records: |  | 0.022 | | -3.8e-3 | -8.4e-3 | -2.5e-5 | 6.6e-3 | 8.3e-5 | NS | NS | 0.97 | 2 | 0.61 |
| assume possibly flu is flu*: |  | 0.021 | | -2.9e-3 | -7.8e-3 | -9.4e-6 | 5.9e-3 | 6.2e-5 | NS | NS | 1.81 | 2 | 0.40 |
| assume possibly flu is not flu: |  | 0.021 | | -3.1e-3 | -8.1e-3 | -1.7e-5 | 5.8e-3 | 7.0e-5 | NS | NS | 1.61 | 2 | 0.45 |
| using highest unit cost for medication*: |  | 0.017 | | -2.1e-3 | -5.7e-3 | -1.6e-5 | 4.5e-3 | 4.6e-5 | NS | NS | 2.60 | 2 | 0.27 |
| excluding outliers#: |  | 0.016 | | -8.0e-5 | -4.3e-3 | NS | 3.1e-3 | NS | NS | NS | 5.60 | 4 | 0.23 |
| excluding censored records: |  | 0.017 | | NS | -3.4e-3 | NS | NS | NS | NS | NS | 4.83 | 6 | 0.57 |
| excluding outpatient records: |  | 0.017 | | -2.5e-3 | -5.9e-3 | -2.2e-5 | 4.7e-3 | 5.5e-5 | NS | NS | 2.52 | 2 | 0.28 |
| assume possibly flu is flu*: |  | 0.017 | | -1.8e-3 | -5.4e-3 | -1.0e-5 | 4.2e-3 | 4.0e-5 | NS | NS | 2.72 | 2 | 0.26 |
| assume possibly flu is not flu*: |  | 0.017 | | -2.0e-3 | -5.7e-3 | -1.6e-5 | 4.3e-3 | 4.6e-5 | NS | NS | 1.80 | 2 | 0.41 |
| Quality-of-life score ambulatory care (normal regression, written responses only) | | | | | | | | | | | | | |
| quality-of-life score: |  | | 0.68 | NS | NS | NA | NS | NA | NA | NA | 3.20 | 3 | 0.36 |
| excluding censored records: |  | | 0.69 | NS | NS | NA | NS | NA | NA | NA | 2.93 | 3 | 0.40 |
| excluding outpatient records: |  | | 0.68 | NS | NS | NA | NS | NA | NA | NA | 3.23 | 3 | 0.36 |
| assume possibly flu is flu: |  | | 0.68 | NS | NS | NA | NS | NA | NA | NA | 3.23 | 3 | 0.36 |
| assume possibly flu is not flu: |  | | 0.68 | NS | NS | NA | NS | NA | NA | NA | 2.98 | 3 | 0.39 |
| Quality-Adjusted Life-Years lost ambulatory care (beta regression (mu) with dispersion (sigma), written responses only) | | | | | | | | | | | | | |
| Quality-adjusted life-years lost: | mu | 3e-3 | | 2e-3 | 6e-3 | 3e-5 | -5e-3 | -7e-6 | -7e-5 | 1e-4 | 0.04 | 1 | 0.84 |
|  | sigma | -3.53 | | 0.49 | NS | 0.008 |  |  |  |  |  |  |  |
| excluding outliers#: | mu | 3e-3 | | 2e-3 | 7e-3 | 2e-5 | -6e-3 | -1e-5 | -9e-5 | 1e-4 | 0.05 | 1 | 0.82 |
|  | sigma | -3.62 | | 0.64 | NS | 0.005 |  |  |  |  |  |  |  |
| excluding censored records: | mu | 1e-3 | | 5e-3 | 6e-3 | 9e-5 | -5e-3 | -1e-6 | -1e-4 | 1e-4 | 0.02 | 1 | 0.90 |
|  | sigma | -2.65 | | 0.20 | NS | -0.43 |  |  |  |  |  |  |  |
| excluding outpatient records: | mu | 3e-3 | | 2e-3 | 6e-3 | 3e-5 | -6e-3 | -7e-6 | -7e-5 | 1e-4 | 0.00 | 1 | 0.99 |
|  | sigma | -3.55 | | 0.49 | 0.008 | NS |  |  |  |  |  |  |  |
| assume possibly flu is flu: | mu | 3e-3 | | 2e-3 | 6e-3 | 3e-5 | -5e-3 | -9e-6 | -6e-5 | 1e-4 | 0.37 | 1 | 0.55 |
|  | sigma | -3.44 | | 0.40 | 0.008 | NS |  |  |  |  |  |  |  |
| assume possibly flu is not flu: | mu | 3e-3 | | 2e-3 | 6e-3 | 3e-5 | -5e-3 | -6e-6 | -7e-5 | 1e-4 | 0.17 | 1 | 0.68 |
|  | sigma | -3.53 | | 0.49 | 0.008 | NS |  |  |  |  |  |  |  |

*mu=mean quality-adjusted life-years ‘s lost; sigma=dispersion parameter, it is used as the covariates have not only an effect on the mean, but also on the variance of the quality-adjusted life-years; ILI=Influenza-Like-Illness; flu= diagnosis (flu or ILI but not flu); cond=underlying condition (no or yes); NS=not significant (p>0.05); NA=not applicable*

*^#^Best fitting distributions: the Gamma for costs of ambulatory patients (for a cost variable, distributions between 0 and infinity are appropriate, the Gamma distribution fitted better than Poisson, Lognormal and Negative Binomial distribution); the Normal distribution fitted well the QoL; and the beta distribution fitted well the QALY’s lost, as they were highly skewed but fell within the 0 to 1 interval.*

*^§^Likelihood ratio test compares model including all (interactions of) covariates with model including significant covariates only.*

**Interaction term flu*age not significant (0.05<p<0.1), but needs to be kept in model based on likelihood ratio test.*

*#Costs: three respondents with costs higher than €400 (lowest cost) are removed; QALY’s lsot: one unlikely flu patients with a very high value of 0.06 is removed, as well as 2 likely flu patients with values higher than 0.027.*
