# Supplementary material for: Influenza-Like-Illness and Clinically Diagnosed Flu: Disease Burden, Costs and Quality of Life for Patients Seeking Ambulatory Care or No Professional Care at All
Source: PLoS One. 2014 Jul 17;9(7):e102634. doi: 10.1371/journal.pone.0102634 (PMC4102549; doi:10.1371/journal.pone.0102634)
Supplement: Supporting Information S1 — Questionnaire used to obtain information on the disease burden, costs and Quality-Adjusted Life-Years of persons who experienced influenza-like-symptoms, in Belgium. This is a translation of the original Dutch and French questionnaires used for completion in writing. In the online and telephone questionnaires, respondents had for each question related to influenza-like-illness the option to answer ‘no answer’ (specified as one or more of the following: don’t remember/not sure/prefer not to answer/no opinion/no answer), because in the online system respondents had to give an answer to be able to continue with the next question. Persons could return to previous questions to specify a different answer. The online system was also used to record answers for the questionnaires completed by phone. (DOCX) [file pone.0102634.s003.docx]

**Influenza-Like-Illness and clinically diagnosed flu: disease burden, costs and Quality of Life for patients seeking ambulatory care or no professional care at all**

Joke BILCKE, Samuel COENEN, Philippe BEUTELS

**SUPPORTING INFORMATION S1: Questionnaire used to obtain information on the disease burden, costs and Quality-Adjusted Life-Years of persons who experienced influenza-like-symptoms, in Belgium.** This is a translation of the original Dutch and French questionnaires used for completion in writing. In the online and telephone questionnaires, respondents had for each question related to influenza-like-illness the option to answer ‘no answer’ (specified as one or more of the following: don’t remember/not sure/prefer not to answer/no opinion/no answer), because in the online system respondents had to give an answer to be able to continue with the next question. Persons could return to previous questions to specify a different answer. The online system was also used to record answers for the questionnaires completed by phone.

The questionnaire consisted of following questions:

1. How many members are there in your household most of the time (including yourself)?
   - Children below 5 years of age: …………….
   - Children from 5 to 12 years: …………….
   - Children from 12 to 18 years: …………….
   - Adults (18 years of older) : …………….
2. Your postcode: …………….
3. Is there someone in your household who experienced flu or a cold in the last four weeks?
   - Yes
   - No

If yes, specify for each person which symptoms they had during the worst episode (if the same person went through several episodes).

If there was more than 1 person in the household, start with the person who became ill as the first in the household.

The respective person …

- is you
- is someone else from your family

Gender:

- male
- female

Is the person an adult or a child?

- adult (18+)
- child

His/her age: ……….. years

Month in which it happened: (several possible)

- December 2011
- January 2012
- February 2012
- March 2012

What were the symptoms of the ill person?

- rapidly rising fever
- high fever (>38°C for an adult and >38.5°C for a child)
- sore throat
- runny or blocked nose
- cough
- sore muscles
- shivering
- nauseous or vomiting
- tired or exhausted

If this person had at least 3 symptoms from the list above, please complete the next questions for this person.

1. How many days did this person had flu-like symptoms during his worst illness episode during the last two months?

Remark: Focus on the worst episode only. Do not sum over several episodes.

Number of days: ………

1. Has this person one or more of these conditions:
   1. Asthma
   2. Diabetes
   3. Heart disease
   4. Other: ………………..
2. If this concerns a women younger than 50 years. Was she pregnant when she had flu-like-illness?
   1. Yes
   2. No

3.A If yes, how many months pregnant: ………..

1. Was this person ever vaccinated against flu?
   1. Yes
   2. No

4.A If yes, when was this person last vaccinated?

- 2012 February
- 2012 January
- 2011 December
- 2011 November
- 2011 October
- 2011 September
- 2010
- 2009
- Before 2009
- Does not know

1. Was a medical doctor consulted for the flu-like symptoms of this person?
   1. Yes
   2. No (in this case, continue with question 7)

If yes, which doctor and how many times?

| Which doctor? | Consult at the doctor’s office (or in the hospital) | Home visits | Consults by telephone |
| --- | --- | --- | --- |
| General practitioner | Number of times: | Number of times: | Number of times: |
| Pediater | Number of times: | Number of times: | Number of times: |
| Pneumologist | Number of times: | Number of times: | Number of times: |
| Otolaryngologist | Number of times: | Number of times: | Number of times: |
| Other specialist | Number of times: | Number of times: | Number of times: |

1. Did the doctor say it was flu, or something else? How did he name it?
   1. flu or influenza
   2. cold or bronchitis,
   3. pharyngitis
   4. otitis
   5. pneumonia
   6. don’t know/remember
   7. other, namely …….
2. Did this person take medication for these flu-like symptoms?
   1. Yes
   2. No (in this case, continue with question 9)

If yes, which medication did this person take?

|  | Taken Yes/No | On prescription Yes/No | Specifically bought (B) or was available at home (H)? |
| --- | --- | --- | --- |
| medication against fever | Yes No | Yes No | B H |
| medication against pain | Yes No | Yes No | B H |
| anti-inflammatory medication | Yes No | Yes No | B H |
| antibiotics | Yes No | Yes No | B H |
| anti-virals (for influenza treatment such as Tamiflu® en Relenza®) | Yes No | Yes No | B H |
| medication against cough (cough sirup,...) | Yes No | Yes No | B H |
| medication against sore throat (sucking tablet, throat spray, …) | Yes No | Yes No | B H |
| nosespray | Yes No | Yes No | B H |
| does not know | Yes No | Yes No | B H |
| other: ................................................. ................................................. | Yes No | Yes No | B H |

1. Can you estimate the medication costs incurred for this person? (so what they had to pay themselves at the pharmacy)
   1. Between 0 and 25 euro
   2. Between 25 and 50 euro
   3. Between 50 and 75 euro
   4. Between 75 and 100 euro
   5. More than 100 euro
   6. Does not know
2. Did this person go to the hospital because of flu?
   1. Yes
   2. No (in this case, continue with question 11)

9.A If yes, to which department did the person go?

- emergency department
- consultation
- intensive care
- lung diseases
- does not know

1. Was this person hospitalized because of these flu-like symptoms? (=at least one night in the hospital)
   1. Yes
   2. No (in this case, continue with question 11)

10.A If yes, for how many days was the person hospitalized?

|  | Number of days in hospital | Of which number of days at intensive care |
| --- | --- | --- |
| Or 1 hospitalization with: |  |  |
| Or several hospitalizations with in total: |  |  |

1. Did this person have to interrupt his/her normal daily activities because of these symptoms?
   1. Yes
   2. No

11.A If yes, how many …

| Days rested |  |
| --- | --- |
| Work days missed |  |
| School days missed |  |
| Days not in childcare |  |

1. Did other persons need to interrupt their normal daily activities in able to care/nurse the sick person? (Mostly relevant for children and elderly)
   1. Yes
   2. No

12.A If yes, how much did these other persons need to interrupt their normal daily activities in order to care/babysit the sick person?

| Number of days family members: |  |
| --- | --- |
| Number of different family members: |  |
| Number of days paid babysitter/nurse: |  |

Did this person recover well?

- 1. Yes, completely recovered
  2. His overall health is weakened.
  3. He is still sick at this moment.
  4. He died because of reasons other than flu.
  5. He died because of the consequences of flu.

1. What is the best description of the job of this person?
   1. Self employed: craftsman, without employees
   2. Self employed: craftsman, with 5 or less employees
   3. Self employed: manager, with 6 or more employees
   4. Self employed: liberal profession
   5. White collar worker: member of the board of directors, higher management
   6. White collar worker: middle management, no member of board of directors
   7. White collar worker: other
   8. Blue collar worker with a professional education
   9. Blue collar worker without a professional education
   10. Housewife/man
   11. Disabled
   12. Retired
   13. Student
   14. Unemployed
   15. Person of independent means

Next, the questionnaire included the Belgium (Dutch or French) SF-12v2 Standard questionnaire (http://www.sf-36.org/demos/SF-12.html).
